# Whole embryo culture, transcriptomics and RNA interference identify TBX1 and FGF11 as novel regulators of limb development in the mouse

**DOI:** 10.1038/s41598-020-60217-w

**Published:** 2020-02-27

**Authors:** Gautier Tejedor, Béryl Laplace-Builhé, Patricia Luz-Crawford, Said Assou, Audrey Barthelaix, Marc Mathieu, Karima Kissa, Christian Jorgensen, Jérôme Collignon, Paul Chuchana, Farida Djouad

**Affiliations:** 1 IRMB, Univ Montpellier, INSERM, Paris, France; 2 CHU Montpellier, Montpellier, France; 3 Laboratorio de Inmunología Celular y Molecular, Facultad de Medicina, Universidad de los Andes, Santiago, Chile; 4 DIMNP, CNRS-UMR5235, Montpellier, France; 5 Université de Paris, CNRS, Institut Jacques Monod, Paris, France

**Keywords:** Apoptosis, Embryology

## Abstract

Identifying genes involved in vertebrate developmental processes and characterizing this involvement are daunting tasks, especially in the mouse where viviparity complicates investigations. Attempting to devise a streamlined approach for this type of study we focused on limb development. We cultured E10.5 and E12.5 embryos and performed transcriptional profiling to track molecular changes in the forelimb bud over a 6-hour time-window. The expression of certain genes was found to diverge rapidly from its normal path, possibly reflecting the activation of a stress-induced response. Others, however, maintained for up to 3 hours dynamic expression profiles similar to those seen *in utero*. Some of these resilient genes were known regulators of limb development. The implication of the others in this process was either unsuspected or unsubstantiated. The localized knockdown of two such genes, *Fgf11* and *Tbx1*, hampered forelimb bud development, providing evidence of their implication. These results show that combining embryo culture, transcriptome analysis and RNA interference could speed up the identification of genes involved in a variety of developmental processes, and the validation of their implication.

## Introduction

A number of studies have attempted to integrate what is known of the genetic and transcriptional control of vertebrate limb formation into temporal gene networks^1–4^. These efforts were based in part on transcriptome data obtained from mouse embryos collected 0.5 or 1 day apart from E9.5 to E13.5^3,5^. This approach identified gene networks tightly regulated during limb morphogenesis as well as novel genes possibly involved in these networks. However, given the complexity and speed of the underlying molecular processes at these stages, relying on time points 1 or even 0.5 day apart limits our ability to capture regulatory changes taking place at a faster pace, and to identify the factors on which they depend.

Whole rodent embryo culture methods have for long allowed the study of aspects of their development that were kept out of reach by viviparity^6^. Embryos explanted after the formation of the placenta and cultured in rolling bottles can develop up to the stage of digit formation on the limbs, an indication that their patterning and differentiation can take place under these conditions. Such conditions, however, are not equivalent to what the maternal environment normally provides, and embryos cultured at these stages are known to have a reduced growth rate, thus resulting in well-formed fetuses that are smaller than normal^7,8^ . So although the culture of whole embryos appears to be a suitable approach to track dynamic molecular changes that underlie early events during limb development, there is a need to assess the extent of its relevance and to define its limits.

We therefore used mouse exon arrays to acquire the global gene expression profiles of developing forelimb buds taken from cultured E10.5 and E12.5 mouse embryos at different time points. We found that the molecular dynamics taking place in cultured embryos very rapidly show signs of diverging from that of embryos developing *in utero*. The analysis and comparison of the different datasets obtained from forelimb buds after 0, 1, 3 and 6 h of culture nevertheless allowed us to discriminate groups of genes for which expression dynamic changes likely reflected developmental progression from others for which these changes resulted from the *in vitro* culture procedure. A number of candidate genes that had not been previously associated with early aspects of limb development were thus identified. Two of them, *Tbx1* and *Fgf11*, were tested for their implication in limb bud development and found to be involved. These results show that although gene expression profiles of cultured embryos quickly diverge from their normal course, their analysis can nevertheless deliver valuable information and lead to a better understanding of actual developmental processes.

## Materials and Methods

### E10.5 and E12.5 mouse embryo culture

Swiss mouse embryos were obtained from the breeding unit of our animal facility (French Health Authorities agreement C34-172-36) and the animals were maintained in accordance with EU directive 2010/63/EU. Since we used mouse embryos at E10.5 and E12.5, a notice of approval from ethics committees is not required. Indeed, under the 2010/63/EU directive, an ethics*-*committee approved animal protocol is needed for mouse embryo from the last third of their gestation or incubation period. Bedding was enriched with wood shaving for nesting and cages were provided with igloos for breeding. Animals used in this study did not receive any treatment. Two females were mate with one male in the late afternoon and vaginal plugs were checked the following morning, considered to be day 0.5 after conception (0.5 dpc). The pregnant mice were euthanized by cervical dislocation, before collecting E10.5 and E12.5 embryos. Experiments were performed in accordance with European and French Agricultural Ministry guidelines for the care and use of laboratory animals (Council directive 2010/63). The procedure for embryo culture was conducted as previously described^9^.

### RNA extraction, array hybridization and data processing

Total RNA was isolated from each sample using RNeasy Mini Kit (Qiagen). The quantity and purity of the total RNA were determined using a NanoDrop ND-1000 spectrophotometer (NanoDrop ND, Thermo Fisher Scientific) and their integrity by using the Agilent 2100 Bioanalyzer (Agilent Technologies; http://agilent.com/). cDNA synthesis, amplification, fragmentation and biotinylation were performed using the Ambion WT Expression Kit (Ambion, Austin, TX, USA). Microarray experiments were performed in the IRMB (Montpellier University Hospital) DNA microarray platform. Samples were hybridized to Affymetrix GeneChip® Mouse Exon 1.0 ST Arrays according to Affymetrix recommendations. These mouse exon arrays consist of an average of 4 probes per exonic region of every primary mRNA transcript allowing to interrogate the entire length of every genome transcript. The experiment was run in triplicate and included the forelimbs of E10.5 mouse embryos, the forelimbs of E10.5 embryos cultured for 1, 3 or 6 hours, the forelimbs of E12.5 mouse embryos and the forelimbs of E12.5 embryos cultured for 1 or 3 hours. Data was acquired on a GeneChip® Scanner 3000 and CEL file generation performed using AGCC. Affymetrix Expression Console™ following standard Exon Array protocols with Robust Multi-chip Average (RMA) was used to extract probe intensity data.

Microarray data were obtained and analyzed in agreement with the minimal information about microarray experiment (MIAME) recommendations^10^. The microarray data were subjected to K-means algorithm and MultiExperiment Viewer (MeV_4_8, version 10.2) software to subdivide the probe set into relevant clusters based on the expression profiles of (i) the forelimb from freshly dissected E10.5 mouse embryos and (ii) the forelimbs from E10.5 and E12.5 mouse embryos cultured for 1, 3 or 6 hours. MeV is a versatile microarray data analysis tool, integrating sophisticated algorithms for clustering^11^. Only transcripts with a significant *p*-value <0.01 were retained. The appropriate workflow for each analysis is described in the corresponding figure. The gene ontology (GO) enrichment analysis, the biological processes and networks of differentially expressed genes were analyzed through the use of IPA (QIAGEN Inc., https://www.qiagenbioinformatics.com/products/ingenuitypathway-analysis)^12^. The Pathway Studio 9.0 software (Ariadne Genomics) was used for the gene interactions and visualization of interactive signaling pathways. Venn diagram are generated using Venny 2.1 Software. All our data are accessible at the gene expression Omnibus (GEO) repository (https://www.ncbi.nlm.nih.gov/geo) with the provisional accession series number GSE117750.

### Flow cytometry analysis (FACS)

For annexin-V and Phospho*-*Histone H3 (pSer^12^) detection, the forelimb bud of embryos at time 0 or cultured for 1, 3 and 6 hours were harvested and incubated for 15 minutes in trypsin, 5% EDTA (Invitrogen). Then, tissues were disrupted mechanically and the released cells washed in complete media. For detection of annexin-V detection, cells were washed with binding buffer and incubated with annexin-V FITC (BD Pharmingen, Le Pont-de-Claix, France) during 15 minutes at room temperature. For Phospho*-*Histone H3 detection, cells were permeabilized with the intranuclear detection Perm/Fix solution (eBioscience) and stained 1 hour with the Phospho*-*Histone H3 (Ser10) antibody (Cell Signaling Technology). Then, cells were washed and stained 30 minutes with anti-rabbit Alexa 647 antibody (Cell Signaling Technology). Finally, samples were acquired on the FACS Canto II and analyzed using the FlowJo software (Ashland, OR, USA).

### SiRNA injection in E9.5 mouse embryos

For *in vivo* transfection reagent studies, 100 μM siRNA solutions were prepared in sterile PBS. Formulation of Injectin (BioCellChallenge) complexes with 100 μM siRNA solutions was prepared according to the manufacturer’s instructions and injected in 4 sites surrounding the left bump on the flank of E9.5 embryos where the forelimb will start to appear at E10.5. For the 4 local injections of 5 nL each, we used the Eppendorf ® FemtoJet® microinjector and then cultured the E9.5 mouse embryos in the roller culture system for 24 hours. We used an untargeted siRNA labeled with the fluorescent dye CY3 (MWG Biotech) both as a control, for the injection - hence its designation as CTL siRNA - and, to track the injection site. It was co-administered with siRNAs against *Fgf11* or *Tbx1* (Ambion) with the siGLO (a non-targeting fluorescent siRNA). The impact of these experiments on the expression of *Fgf11* and *Tbx1* was monitored by RT-qPCR and whole mount *in situ* hybridization (WISH).

### RT-qPCR analysis

Total RNA was extracted using the RNeasy mini kit (Qiagen S.A.). RNA (500 ng) was reverse transcribed using the Multiscribe reverse transcriptase (Applied Biosystems). Quantitative PCR was performed using the SYBR Green I Master kit and a LightCycler^®^ 480 Detection system, following manufacturer’s recommendations (Roche Applied Science). Specific primers for *Fgf11* and *Tbx1* were designed using the Primer3 software (Fgf11 F: TCC TCA TCC TGC TGT CCA AGG T; Fgf11 R: ATT CGC CTG GAG GTA GAA ACC C; Tbx1 F: CGA GAT GAT CGT CAC CAA GGC A; Tbx1 R: GTC ATC TAC GGG CAC AAA GTC C. Data were normalized to the housekeeping gene ribosomal protein S9 (RPS9). Values were expressed as relative mRNA level of specific gene expression as obtained using the 2^−ΔCt^ method.

### Whole mount *in situ* hybridization

*Fgf11*, *Tbx1* and *Sox6* antisense RNA probes were generated from cDNA clones (*Fgf11* clone: 30101881/IRAVp968D07109D, *Tbx1* clone: 8862496/IRCKp5014O0612Q, *Sox6* clone: 30094413/IRAVp968C07156D- Life Sciences Source BioScience), and Digoxygenin (DIG) labeled using a Dig labelling kit (Roche). Whole mount *in situ* hybridization were performed on E10.5, 24 hours-cultured E9.5 mouse embryos as previously described^13^. Finally, images were acquired on Olympus MVX10 (Supplementary Fig. S3A) or Zeiss SteREO Discovery.V12 (Supplementary Fig. S3B) stereo microscopes.

### Monitoring of forelimb development of E9.5 mouse embryos after siRNA injection and culture

Left forelimb development was monitored by measuring the surface of the left forelimb of E9.5 mouse embryos cultured for 24 hours (beginning of the bump on the flank) up to the most proximal end of the forelimb. To exclude the size variability between mouse embryos and quantify the effect of the siRNA injection on the development of the left forelimb, the measured area of the left forelimb was normalized by the surface of the untreated right forelimb of mouse embryos. The measure of the forelimb surface was done with the ImageJ software. Significance testing was done using Mann-Whitney unpaired t-test, two tails using GraphPad Prism 6 Software. Graphs show mean ±Standard Error of the Mean (SEM).

### Statistical analysis

Results are expressed as the mean ± SEM and all experiments were performed at least 3 times. Generated P values were obtained using Mann-Whitney unpaired t-test, two tails using GraphPad Prism 6 Software. Graphs show mean ±Standard Error of the Mean (SEM). P-values <0.05 (*), P < 0.01 (**) or P < 0.001 (***) were considered statistically significant. Analysis and graphical representation were performed using Graph-Pad Prism^TM^ software (Graphpad).

## Results

### Extended embryo culture delays developmental progression

**Figure 1:**
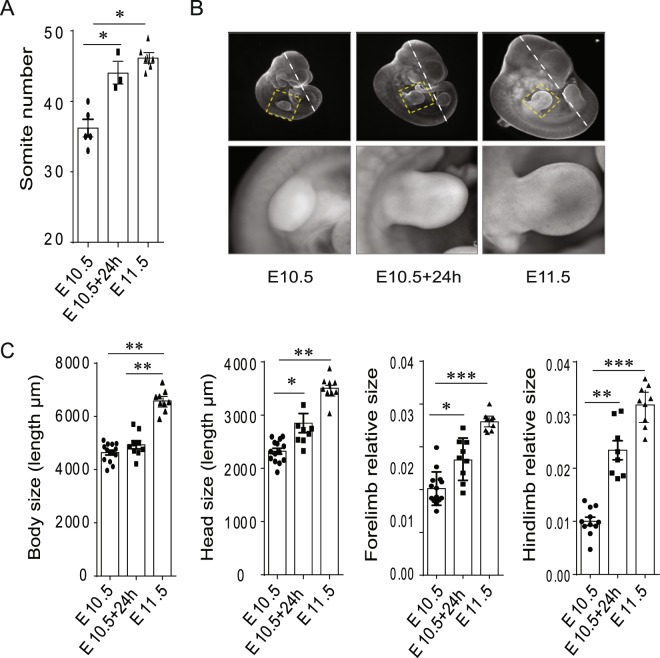
Cultured E10.5 mouse embryos exhibit evidence of a developmental delay. (**A**) Quantification of the total somite number in uncultured E10.5 and E11.5 mouse embryos and in *ex utero* developing E10.5 mouse embryos during 24 hours. (**B**) Pictures of the right forelimb of uncultured, 24 h *ex utero* cultured E10.5 and uncultured E11.5 mouse embryos. (**C**) Assessment of body size corresponding to the Crown to Rump length (hatched white line traced in panel B), head size in µm and the relative limb size (forelimb and hindlimb) of *ex utero* and *in utero* developing embryos. The relative sizes of the forelimb and hindlimb correspond to their respective surface normalized by the body size of the embryo. Error bars indicate standard error of the mean ( ± SEM).

E10.5 mouse embryos were cultured in rotating bottles with constant gassing for 24 hours^14^. Frequent monitoring of their heartbeat showed that they remained alive for the duration of the culture. They visibly gained in size and in vascular complexity, indications that the procedure did not prevent them from furthering their development. To assess what developmental stage they had reached after 24 h culture we compared their total somite number to that of *in utero*-developed E11.5 embryos. This number increased from 32–40 to 42–47 in cultured embryos, a final tally similar to the 45–47 somites counted in E11.5 embryos (Fig. 1A), suggesting that the pace of development was barely affected by culture conditions. However, compared to E11.5 embryos, cultured embryos exhibited a noticeable overall growth delay (Fig. 1B,C). Focusing on the development of the head and the limbs (forelimb and hindlimb), we noticed a significant increase of their size in cultured embryos, but this increase was only half of that measured in E11.5 embryos (Fig. 1C). In contrast the same cultured embryos showed no increase in body size. These results indicate that, after 24 hours, although this culture system allows E10.5 embryos to be kept alive for extended period of times and their development to proceed, their growth is clearly affected, indicating that culture conditions are not optimal. They also show that different body parts are affected differently, suggesting that the developmental program adapts to suboptimal conditions, sustaining growth less in some regions than in others. In this context limb development provides a suitable benchmark to assess the extent of this growth defect and its impact on development. We decided to investigate the establishment of this defect and thus to determine to what extent *in vitro* development is relevant to the study of developmental events taking place *in utero*. Defining the limits of *in vitro* culture approaches is an important issue given their widespread use.

### Gene expression dynamics in forelimb buds developing ex utero

**Figure 2:**
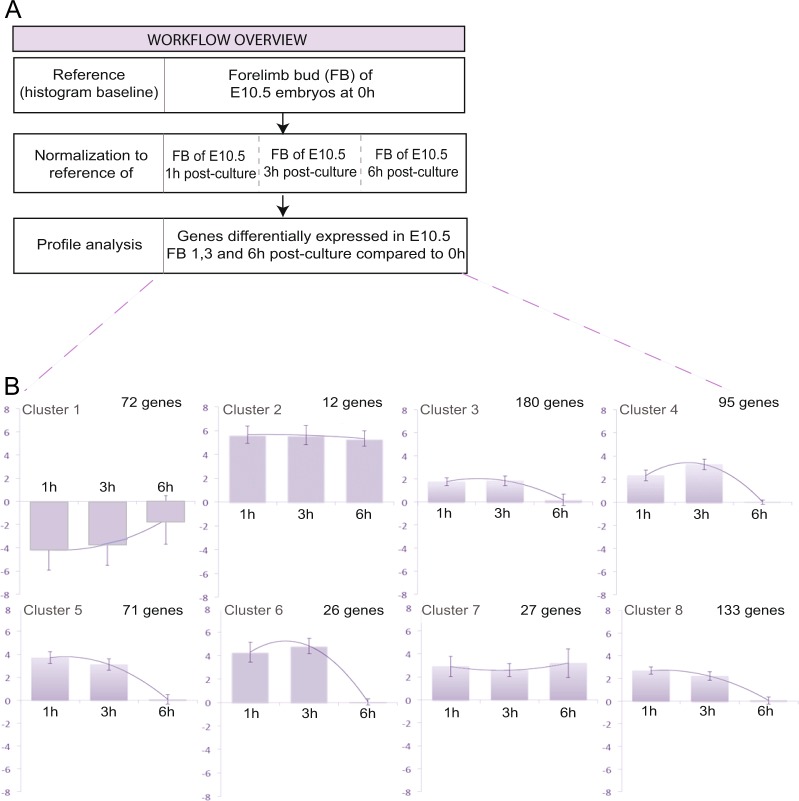
Gene expression dynamics in forelimb buds developing *ex utero*. (**A**) Overview of approach and computational analysis. We used the forelimb bud of E10.5 embryos at time 0 as a reference and attributed the value 0 to all the genes expressed in this sample of reference (Reference, Histogram baseline). The relative expression level of genes differentially expressed during the *ex utero* development of the FB of E10.5 mouse embryos after 1 h, 3 h and 6 h of culture, is normalized to reference genes expressed in FBs of freshly dissected E10.5 embryos (histogram baseline). (**B**) Expression profile of genes differentially regulated during the *ex utero* forelimb bud development of E10.5 mouse embryos. After 1 and 3 hours of culture we identified 616 genes with a linear dynamic expression through the time course of *ex utero* development. However, most of these genes saw their expression going back to its original level after 6 hours of culture. The Y axis represents a normalized expression variation.

To define the time window during which *ex utero* development faithfully reflects *in utero* development at the molecular level, we performed transcriptome analyses of forelimb buds (FB) from cultured E10.5 embryos to identify and characterize differentially regulated gene sets. RNAs were extracted from the FBs of E10.5 embryos either freshly dissected or after 1, 3 or 6 hours of culture in rotating bottles. They were analyzed using the Affymetrix GeneChip Mouse Exon 1.0 ST microarray, that provides a complete expression profile of messenger RNA (mRNA) as well as the intermediary long intergenic non-coding RNA (lincRNA). For all genes thus found expressed, their expression level at E10.5 (or time 0) was set at 0 (histogram baseline), and used as a reference for comparison with their expression levels in cultured embryos (Fig. 2A). Using Multi-Experiment Viewer software and K-means algorithm, we identified differentially expressed genes that could be grouped into eight clusters according to their respective expression dynamics over the 6-hour time course (Fig. 2B and Supplementary Table 1). For six of these clusters, the gene expression profiles went back to their original level after just 6 hours of culture. For the other two, gene expression went up to a certain level within the first hour of culture and maintained this level for the rest of the time-course (Fig. 2B and Supplementary Table 1). These observations indicate that culture conditions affect developmental dynamics quite rapidly.

### Extended embryo culture triggers stress-induced cellular response

Are the gene expression dynamics we observed the result of a culture-induced stress? A cell’s response to stress involves a choice between death and survival, with competing processes leading either to apoptosis or to cell dedifferentiation and proliferation^15^. We therefore investigated whether any of these cellular processes was affected in cultured embryos.

**Figure 3:**
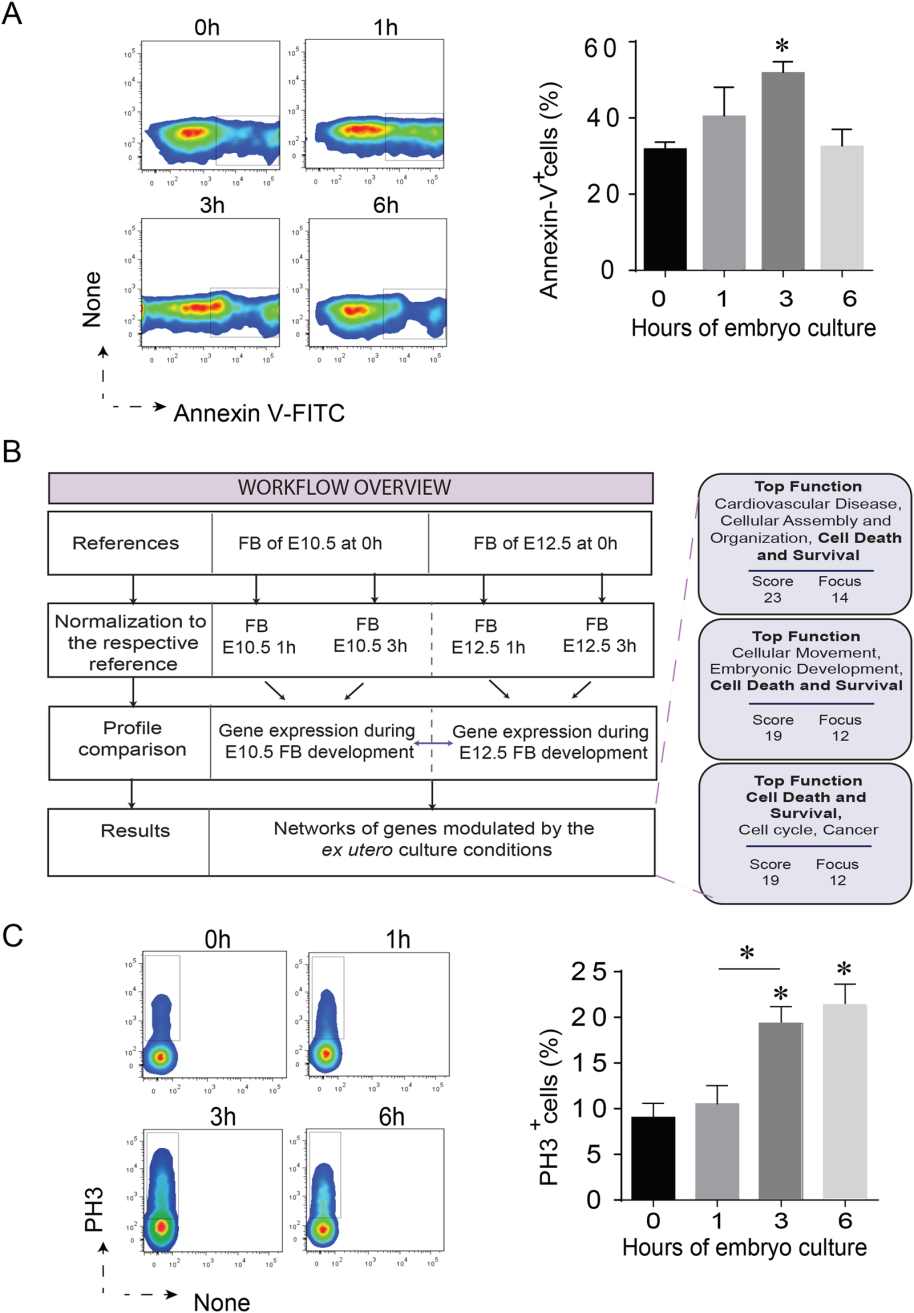
Impact of embryo culture on tissue growth. (**A**) Percentage of Annexin-V positive cells in the forelimb bud of E10.5 mouse embryos before *ex utero* culture and after 1, 3 and 6 h of culture. Results of the FACS analysis are represented in the right panel as histogram (%± SEM). In the left panel, a representative FACS dot plot of Annexin-V + cells at each time point for one mouse embryo. (**B**) Overview of approach and computational analysis. The functional analyses were generated through the use of IPA (QIAGEN Inc., https://www.qiagenbioinformatics.com/products/ingenuity-pathway-analysis). During the first hours of mouse embryo culture in a controlled system, 39 out of 83 genes display a similar expression dynamic and are associated with cell death and survival processes. (**C**) Percentage of H3 phosS10 positive cells in the forelimb bud of E10.5 mouse embryos before *ex utero* culture and after 1, 3 and 6 h of culture. Results of the FACS analysis are represented in the right panel as histogram (%± SEM). In the left panel, a representative FACS dot plot of H3 phosS10^+^ cells at each time point for one mouse embryo.

First, FACS analysis of FBs stained with FITC-conjugated Annexin-V allowed us to detect a peak of apoptotic cells in the buds of E10.5 embryos cultured for 3 hours. The percentage of Annexin-V-positive cells then reverted to E10.5 reference level after 6 hours of culture (Fig. 3A).

Second, to identify genes that might be specifically involved in the response to culture conditions we looked for genes that displayed similar expression dynamics during the first 3 hours of culture when starting with embryos taken at two different embryonic stages: E10.5 and E12.5. To do so, we performed transcriptome analysis as described before on FBs taken from E12.5 embryos, either freshly dissected or after 1 or 3 hours of culture. Analysis and comparison of the E12.5 datasets with their E10.5 counterparts allowed us to identify 83 genes showing a similar expression profile over the 3-hour time course (Fig. 3B, Supplementary Table 2). The expression of these genes thus appears to be modulated by the procedure undergone by the embryos, including their harvesting and culture, not by stage-specific developmental processes. These 83 genes were then categorized based on their functions, interconnectivity, and involvement in canonical pathways using the Ingenuity Pathway Analysis application (IPA) (Fig. 3B, Supplementary Fig. S1). The genes were mapped in the Ingenuity Pathways Knowledge Base (IPKB) with their respective expression values and designated focus genes. Based on gene connectivity, the algorithm generated networks of focus genes and assigned them a score taking into account the number of focus genes and the size of each network (Fig. 3B, Supplementary Fig. S1). Thus, IPA analysis of the 83 genes found that 38 of them were implicated in gene networks associated with cell death and survival (Fig. 3B, Supplementary Fig. S1, Supplementary Table 2), suggesting that the *ex-utero* embryo culture procedure triggers a specific response from these gene networks.

We then investigated whether cell proliferation is also affected in cultured embryos. To that end, we quantified histone H3 phosphorylation at Ser10, a recognized marker of mitotic chromosomes^16,17^, and found a significant increase in the FBs of cultured mouse embryos at the 3-hour mark, still maintained after 6 hours of culture (Fig. 3C).

Taken together, our results indicate that placing embryos in culture rapidly triggers a complex stress-induced response, which can be detected in FBs and involves the activation of several gene networks, including some associated with apoptosis and proliferation (Supplementary Fig. S1).

### Validation of ex utero culture conditions

**Figure 4:**
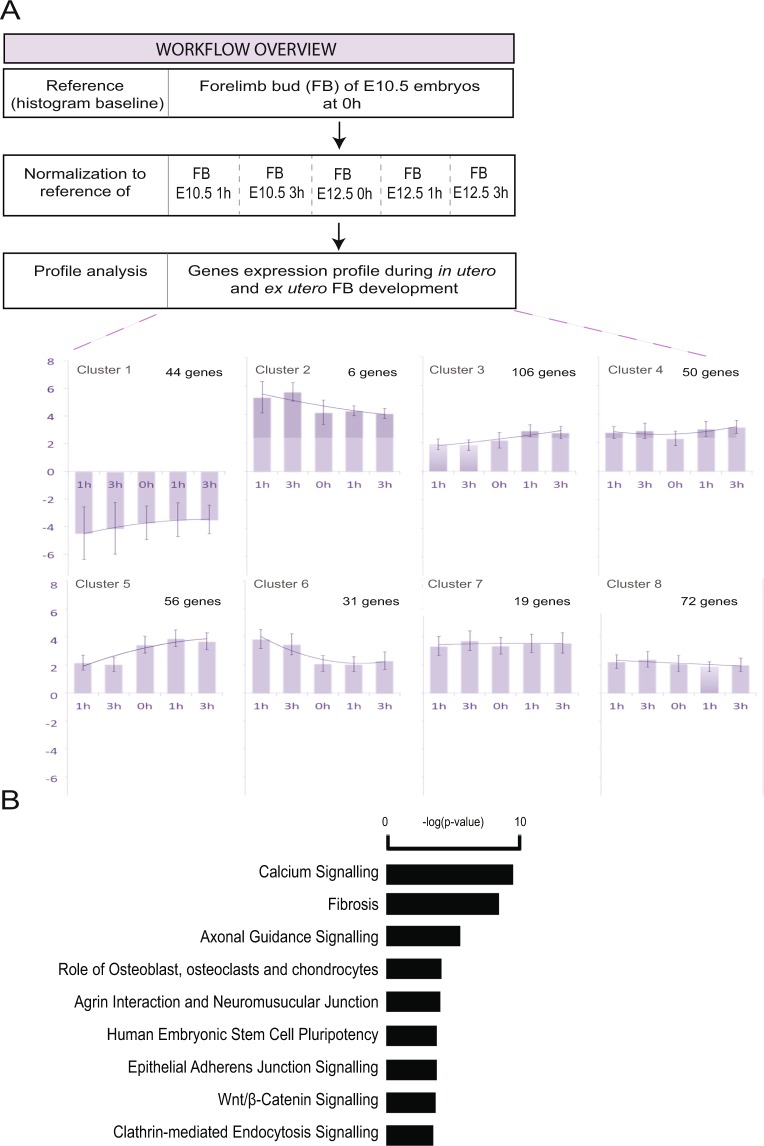
Genes differentially expressed in *ex utero* and *in utero* developing forelimb buds. (**A**) Overview of approach and computational analysis. Expression profiles of genes differentially regulated during the *ex utero and in utero* forelimb bud development of E10.5 and E12.5 mouse embryos. The forelimb bud of E10.5 embryos at time 0 is used as a reference and the value 0 was attributed to all the genes expressed in this sample of reference. After 1 and 3 hours of culture of E10.5 and E12.5 embryos and in the forelimb of non-cultured E12.5 embryos we identified 675 probe sets with a linear dynamic expression through the time course of *ex utero* and *in utero* forelimb development. The Y axis represents a normalized expression variation. (**B**) Genes differentially expressed in the forelimb of *in utero* and *ex utero* developing embryos were associated with mainly 9 distinct different canonical pathways identified by IPA (*P* < 0.05). The *y*-axis represents the −log of *P*-values calculated by Fischer exact test.

We then wanted to assess how long embryos developing in rolling bottles remain an informative *in vitro* model when studying molecular events surrounding forelimb development. Using gene expression levels in the FBs of freshly dissected E10.5 embryos as a reference, we compared them to those found in E10.5 FBs after 1 hour or 3 hours of embryo culture, as well as to those of E12.5 FBs, either freshly dissected or after 1 hour or 3 hours of embryo culture. The analysis of gene expression dynamics allowed us to group 273 differentially expressed genes into 8 distinct gene clusters using Multi-Experiment Viewer software and K-means algorithm (Fig. 4A and Supplementary Table 3). In most of these, gene expression dynamics showed some continuity between the values obtained in E.10.5 and E12.5 FBs. This suggests that cultured embryo FBs maintain expression levels resembling those of *in utero* developing FBs for at least 3 hours. To identify the processes and cellular components with which the differentially expressed genes we identified in developing FBs associate, we used the Gene Ontology (GO) resource to identify enriched GO terms (Supplementary Fig. S1). Collagen, the main structural component of the connective tissues was the term ranked first in this analysis, followed by calcium channel complex and voltage-gated calcium channel complex, actin filament and actin cytoskeleton, striated muscle thin filament and sarcomere (Supplementary Fig. S1). The same genes were also analyzed using the IPA application to identify the biological and physiological processes with which their gene networks associated. The terms that came out of this analysis, starting with calcium signaling, fibrosis and axonal guidance signaling, were consistent not only with our previous GO term analysis, but also more generally with the molecular and cellular processes known to take place during early limb development (Fig. 4B). Finally, comparison with the list of 83 genes identified as being dependent on cell culture conditions revealed that 50 of them, including 21 associated with cell death and survival networks, were also on the list of the 273 differentially expressed genes (Supplementary Table 4).

### Ranking novel regulators for forelimb development

**Figure 5:**
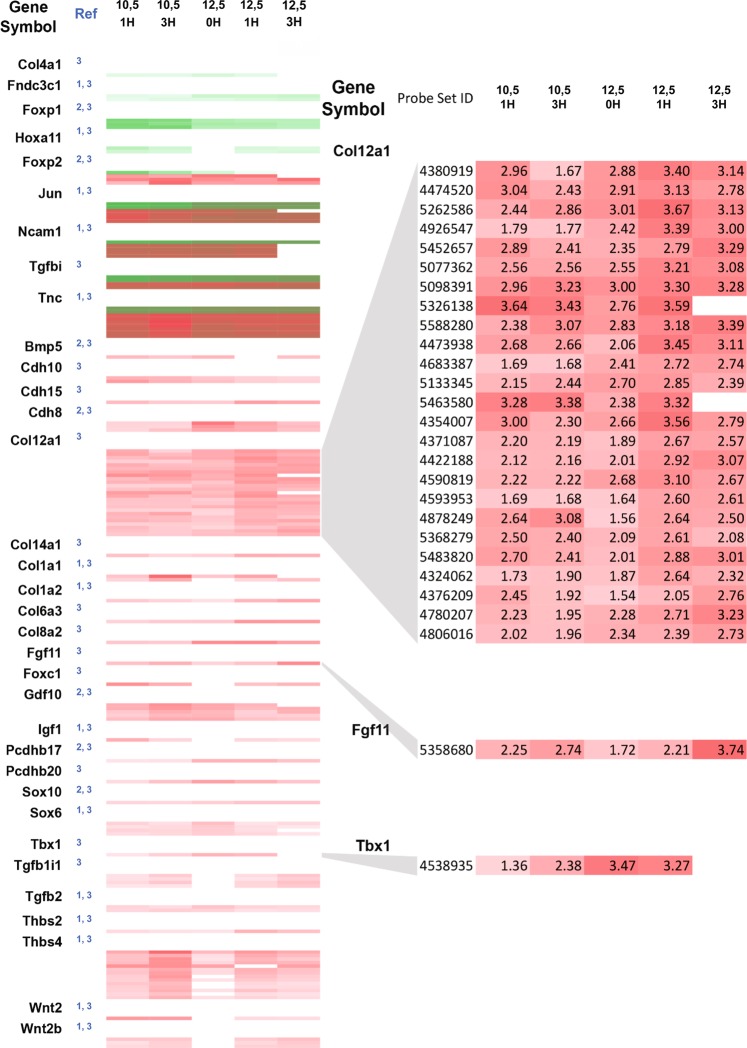
Gene expression profiles in *ex utero* and *in utero* developing forelimbs of E10.5 mouse embryos. Shown are the differential expression levels for probe sets (rows) at 5 time points (columns) in the *ex utero* developing limb of E10.5 and E12.5 embryos after 1 and 3 hours of culture and non-cultured E12.5 embryos relative to control non-cultured forelimb of E10.5 mouse embryos. Genes listed on the left are all targeted by several probe sets, revealing information about the similarity and discrepancies of each alternative probe set pair. In green are represented the probe sets down regulated and in red the probe sets overexpressed in the developing forelimb as compared to the forelimb of E10.5 mouse embryos at time 0. Some probe sets that interrogate different regions of *Jun, Ncam1, Tgfbi and Tnc* are either up- or down-regulated as compared to the reference sample while all the probe sets that target the other genes of the heat map are all regulated in the same manner for one given gene. We compared the genes differentially expressed during embryo cultures (present study referred as 3 in blue in the column “Ref”) with genes described in the literature to be differentially regulated during in utero limb development (referred as 1 and 2). References 1 and 2  correspond to a review dissecting the gene networks involved in the control of chondrogenesis^4^, and to a study providing a global gene expression profiling analysis of mouse embryo limb during *in vivo* development from 11.5 dpc to 13.5 dpc^3^, respectively.

To further validate the embryo culture approach we looked in our data set at the expression dynamics of 21 genes previously shown to be differentially regulated during the development of the forelimb in mouse^3,4^. For 4 of them the data was inconclusive, distinct probe sets giving conflicting results. However, the remaining 17 were differentially expressed over the time-course considered, with 3 of them down-regulated and 14 up-regulated with reference to the initial expression level in the FB of freshly dissected E10.5 embryos (Fig. 5). This confirmed that the information in the expression data sets obtained from culture embryos is relevant and informative with regard to *in utero* development. We then looked in our data sets for differentially expressed genes that were not previously shown to be involved in limb development, but belong to gene families that include at least one member that is known to be involved. We identified 13 such genes. *Col12a1* is one of them, a result supported in this case by the 25 relevant probe sets all detecting a similar expression dynamic (Fig. 5). Is such a differential expression, detected in the FBs of cultured embryos, predictive of an implication in limb development?

### Ex utero investigation of the role of known regulators of forelimb development

**Figure 6:**
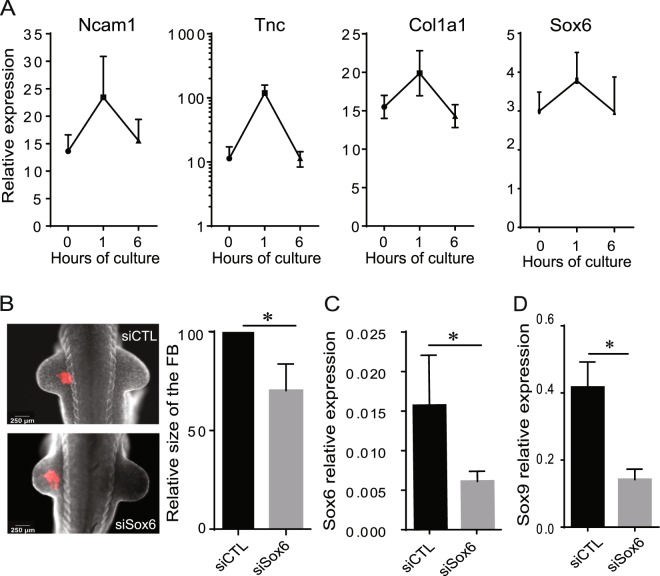
*Ex utero* investigation of *Sox6* implication in forelimb development. (**A**) Gene expression levels of *Ncam1*, *Tnc*, *Col1a1* and *Sox6* in the FBs of *ex utero* developing embryos after 0, 1 and 6 hours of culture. (**B**) E10.5 mouse embryos co-injected with the siCTL tagged with CY3 and siRNA targeting Sox6 or the two simultaneously in the left forelimb of E9.5 embryos cultured for 24 h in the roller culture system were imaged using a Zeiss Discovery V8 fluorescence stereomicroscope. Measure of the surface of the left forelimb of E9.5 mouse embryos cultured 24 h after siRNA administration normalized by the surface of the untreated right forelimb of mouse embryos. The measure of the forelimb contour was done with the ImageJ software. (**C**) Relative expression profile of *Sox6* in the forelimb of mouse embryos that have been transfected at E9.5 either with the control siRNA (siCTL) or with the siRNA targeting *Sox6* (siSox6), and cultured for 24 h. (**D**) Relative expression profile of *Sox9* in the forelimb of E10.5 mouse embryos that have been transfected with either the siRNA control (siRNA CTL) or the siRNA against *Sox6* at the embryonic stage E9.5 and cultured 24 h *ex utero*. Each graph represents the mean value of at least 3 independent experiments ± SEM. *P-values <0.05 (*).

First, we assessed by RT-qPCR the expression dynamics of *Ncam1*, *Tnc*, *Col1A1* and *Sox6*, which were all previously shown to be differentially regulated during forelimb development in the mouse^3,4^, and identified as such in our transcriptome analysis (Fig. 5). RT-qPCR analysis showed that all four exhibited increased expression in the FBs of E10.5 embryos after just 1 hour of culture (Fig. 6A). Of note, each of these genes belongs to at least one of the clusters of genes whose expression after 6 h of culture goes back to the level at which it started (Fig. 2B, Supplementary Table 1). This loss of differential expression level was confirmed by RT-qPCR for the 4 tested genes, thus validating the transcriptome analysis (Fig. 6A).

Then, we focused our attention on the role of *Sox6* whose differential expression during the *ex utero* development of the forelimb was conclusive from the transcriptome analysis (Fig. 5) and validated by RT-qPCR (Fig. 6A). *Sox6*, which we found up-regulated in the FB of E10.5 embryos after just 1 hour in culture, is already known to be implicated in limb development*. Sox6* is induced together with *L-Sox5* after the expression of *Bmpr1*, which precedes the onset of morphological modifications in pre-chondrogenic aggregates, and this induction is associated with that of *Col2* and *Aggrecan* in the forming cartilage^18,19^. We used a loss-of-function approach to test the implication of *Sox6* in FB development. Briefly, we mixed a lipid-based *in vivo* transfection reagent (Injectin) with a siRNA targeting *Sox6* (Sox6-siRNA), and injected the solution at 4 sites surrounding the future FB on the flank of E9.5 embryos prior to culturing them for 24 hours. This siRNA was co-injected with an untargeted siRNA carrying a CY3 fluorescent tag to localize the injection sites. This fluorescent untargeted siRNA (CTL-siRNA) also provided the control condition when injected on its own. After 24 hours of culture we observed a normal development of the FBs injected with the CTL-siRNA alone (Fig. 6B). In contrast, the development of FBs co-injected with the Sox6-siRNA was significantly impaired (Fig. 6B). RT-qPCR and whole-mount *in situ* hybridization (WISH) showed that this correlated with a decrease of *Sox6* in these FBs, attesting of the efficiency of the siRNA (Fig. 6C, Supplementary Fig. S2B). Furthermore, these FBs also showed a drastic reduction in the expression of *Sox9*, another known regulator of FB development (Fig. 6D).

These results validated our approach to assess the implication of candidate genes in limb development.

### Identification of novel regulators involved in forelimb development

Next, we used this approach to investigate the implication of genes we identified as potential regulators of limb development in our transcriptome analysis. We picked two genes, *Fgf11* and *Tbx1*, respectively from clusters 4 and 5 (Fig. 4A and Supplementary Table 3), that the transcriptome analysis found upregulated in developing FBs (Fig. 5). *Fgf11* belongs to the *Fgf* gene family^20^, some members of which are expressed in the developing limb and contribute to its development^21,22^. The fact that a previous transcriptome analysis of E12.5 limb buds detected *Fgf11* expression^21^ but that no evidence linked it to limb development so far made it an interesting candidate for us to pursue. *Tbx1* is a member of a gene family encoding transcription factors, some of which are known to participate in limb development^23^*. Tbx1* expression was previously characterized in E12.5 limb buds^21^, but the study of *Tbx1*^−/−^ embryos found no defect in the development of their limb^24^. We wondered whether our siRNA-based approach might elicit a different result given recent evidence that knockdown experiments are less susceptible to genetic compensation than knockout experiments^25^.

**Figure 7:**
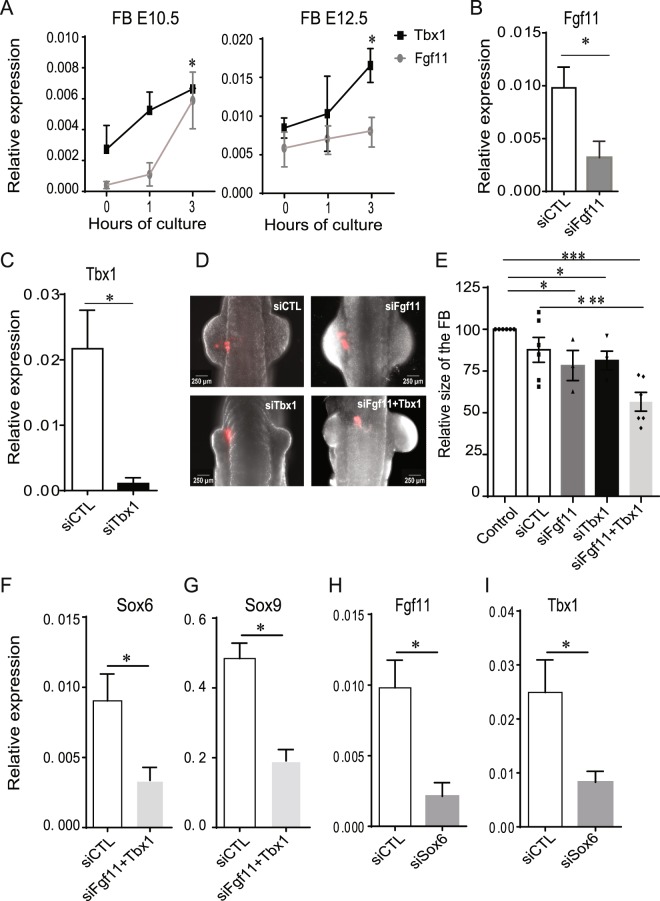
Investigation of *Fgf11* and *Tbx1* implication in forelimb development. (**A**) Relative expression profiles of *Fgf11* and *Tbx1* in the forelimb of E10.5 (left) and E12.5 (right) mouse embryos after 1 and 3 hours of culture. RT-qPCR on separated cells using *Rps9* as a reference gene. (**B-C**) Relative expression profiles of *Fgf11* and *Tbx1* in the forelimb of E10.5 mouse embryos that have been transfected with either the siRNA control (siRNA CTL) or the siRNA against *Ffg11* or *Tbx1* (si*Fgf11* or si*Tbx1*, respectively) at the embryonic stage E9.5 and cultured 24 h *ex utero*. (**D**) E10.5 mouse embryos co-injected with the siCTL tagged with CY3 and siRNA targeting either *Fgf11* or *Tbx1* or the two simultaneously in the left forelimb of E9.5 embryos cultured for 24 h in the roller culture system were imaged using a Zeiss Discovery V8 fluorescence stereomicroscope. (**E**) Measurements of the surface of the left forelimb of E9.5 mouse embryos cultured 24 h after siRNA administration normalized by the surface of the untreated right forelimb of mouse embryos. (**F-G**) Relative expression levels of *Sox6* and *Sox9* in the FBs of *ex utero* developing embryos 24 h after the transfection with siRNA against *Fgf11* or *Tbx1*. (**H-I**) Relative expression levels of *Ffg11* (**H**) and *Tbx1* (**I**) in the forelimb of E10.5 mouse embryos that have been transfected with either the siRNA control (siRNA CTL) or the siRNA against *Sox6* at the embryonic stage E9.5 and cultured 24 h *ex utero*. Each graph represents the mean value of at least 3 independent experiments ± SEM. *P-values <0.05 (*) or P < 0.001 (***).

RT-qPCR confirmed that the expression of *Fgf11* and *Tbx1* increased significantly between E10.5 and 12.5 in the FBs of cultured embryos (Fig. 7A). *In situ* hybridization revealed that the expression patterns of *Fgf11* and *Tbx1* in the FB of E10.5 embryos were similar to that of *Sox6* (Supplementary Fig. S2A). To test possible requirements for *Fgf11* and *Tbx1* during forelimb development, E9.5 mouse embryos were injected as before with siRNAs targeting either one or the other. RT-qPCR analysis after 24 h culture showed that these siRNAs significantly decreased the expression levels of their targets in the FBs of injected embryos when compared to the effect of the CTL siRNA (Fig. 7B,C). The knockdown of *Fgf11* or *Tbx1* also resulted in a downregulation of *Tbx1* or *Fgf11* expression, respectively, suggesting some interdependency in their regulation (Supplementary Fig. S3C). However, unlike with the *Sox6* knockdown, *in situ* hybridizations did not show a difference in the expression of *Fgf11* or *Tbx1* in embryos where they had been targeted (Supplementary Fig. S2B). The knockdown of either gene nevertheless had an impact on the development of the limb bud. It was assessed by measuring the surface of the injected FB relative to that of its uninjected counterpart. While the CTL siRNA had a limited impact, the injection of Fgf11- or Tbx1-siRNAs resulted in limb buds that were 20 to 25% smaller than controls, a result consistent with the implication of these genes in limb bud development. The co-injection of the Fgf11- and Tbx1-siRNAs resulted in a dramatic 45% reduction in the size of FBs, suggesting that the two genes act in a synergistic manner (Fig. 7D,E). Moreover, this effect was associated with a significant decrease in the expression of *Sox6* and *Sox9*, known master regulators of FB development (Fig. 7F,G). This enticed us to look at the impact of the knock-down of *Sox6* on the expression of *Fgf11* and *Tbx1*. Interestingly, we found that the expression of both genes was greatly reduced (Fig. 7H,I), suggesting *Sox6*, *Sox9*, *Fgf11* and *Tbx1* operate within the same regulatory network in the developing FB.

## Discussion

Analysis of the transcriptomes of FBs from cultured E10.5 and E12.5 embryos, and comparison with those from their freshly dissected counterparts, allowed us to distinguish over a short time-window changes in gene expression that still reflected ongoing developmental process as they normally take place *in utero*, from changes in gene expression that were dependent on a stress-induced response triggered by the adaptation of the embryos to culture conditions. We found that genes already known to be differentially expressed during FB development were also likely to be correctly identified as such in our analysis. We set up and validated an approach to test the implication of candidate genes in FB development, relying on the local knockdown of their expression via lipofection of specific siRNAs. We then used it to assess the implication of *Fgf11* and *Tbx1*, two of the genes that our analysis identified as differentially expressed during this process, but that had not so far been associated with it. We found that the down-regulation of their expression disrupted regulatory networks known to govern forelimb development and resulted in stunted buds. These results suggest the approach we have delineated, combining short-term embryo culture, transcriptome analysis and RNA interference, could speed up the identification of candidate genes potentially regulating the development of various embryonic or fetal structures, and the validation and characterization of their implication. It provides a sensible alternative to the use of organotypic cultures.

Mouse embryo culture methods are well established. They provide an imperfect but practical system to study developmental processes that are otherwise hidden from view. When performed at stages that cover the early steps of organogenesis, the rolling bottle method we used in this study can allow development to proceed at a pace close to that seen *in utero* for up to 24–36 h. At later stages however, its capacity to support embryonic development is greatly reduced, presumably because there is no flow of culture medium equivalent to a maternal blood circulation running through the placenta, which by then has formed. We noticed, that although E10.5 embryos cultured for 24 h formed the expected number of somite pairs, indicating that they had reached a developmental stage equivalent to E11.5, their body size remained unchanged. Head and limb bud measurements also showed their gain in size was about half of what was expected. These defects are reminiscent of the impact of under-nutrition during gestation, which has been associated with neonates exhibiting a normal head size on a relatively small body^26^. Such defects were proposed to result from an adaptive response of the foetus to unfavourable nutritional conditions, a response that protects brain development at the expense of that of the trunk. Prioritizing the development of certain body parts over others to mitigate challenging developmental conditions is seen as a possible origin for an array of disease that may develop later in life. Neonates with reduced abdominal circumferences are for example more likely to develop heart diseases^26–28^. This appears to be consistent with our finding that the highest-scoring network of genes differentially modulated by culture conditions is linked to cardiovascular disease, cellular assembly and organization and cell death and survival. All of this strongly suggests that the defects seen in cultured embryos primarily result from their under-nourishment, and from the adaptive response it elicits.

3 hours after E10.5 embryos were put in culture we detected a peak of apoptosis in their FBs. The rate of mitosis was also significantly increased after 3 hours, and kept increasing during the following 3 hours. This is consistent with the script followed by a stress-induced response, which usually entails apoptosis, dedifferentiation and proliferation^29,30^. To identify genes that might be involved in or dependent on the stress-induced response we postulated that culture conditions would have the same effect on their expression profile at E12.5 than at E10.5. 83 differentially expressed genes were found to fit this description, and nearly half of them turned out to be associated with cell death and survival networks. The response to the dissection and culture conditions is therefore swift and strong and appears to be triggered very rapidly, increase in the expression of these genes being detected after just 1 hour of culture. This suggests that the whole embryo culture procedure affects molecular processes in the embryo as soon as it begins, and that culture conditions are never optimal.

In these conditions, how long were the expression profiles of developmental genes similar to what they are *in utero*? Our analysis revealed that although the expression levels of most differentially expressed genes in the FBs of cultured E10.5 embryos went back to where they started after 6 hours of culture, 3 hours earlier they were closer to what they normally are at E12.5. This suggests that although culture conditions are stressful for the embryo and trigger a specific response, they can nevertheless sustain the regulation of gene expression that normally underlies FB development for 3 hours. 273 developmentally regulated genes were thus identified. Interestingly, 50 of these genes (18%), including 21 associated with cell death and survival networks, were also listed among the 83 that had been previously recognized as potentially dependent on the stress-induced response. This meant that our criteria for the identification of developmentally regulated genes led to the rejection of 33 differentially expressed genes, but also to the inclusion of 50 that may in fact depend on culture conditions.

Our criteria to identify potential developmental regulators and genes affected by culture conditions are not mutually exclusive and are bound to select candidates that end up on both lists. This may be legitimate, as there are multiple examples of genes involved in stress-induced response or cell death and survival networks that also participate in normal developmental processes. However, it is also possible that our criteria were not stringent enough to prevent the inclusion on our list of potential developmental regulators of genes that play no such role. The evaluation of the 50 candidate genes common to both lists will thus require an additional layer of scrutiny. The GO terms, gene networks and processes our 273 candidates were found associated with were nevertheless consistent with what early limb development involves. The fact that out of 21 genes known to be differentially expressed during limb development^3,4^ 17 (81%) were among the 273 identified in our analysis attest of the power of our approach. However, to apply it to the identification of developmentally regulated genes in other organs or body parts may require further characterization. Since culture conditions do not have the same impact on the development of all body parts the time window during which transcriptome analysis remains informative may vary accordingly.

To test the implication in limb development of the genes we identified we aimed to develop an approach that was fast and efficient. The local knock-down of the expression of candidate genes via lipofection of specific siRNAs in FBs proved to be both when we tested it on *Sox6*. We could validate its impact on the expression of the gene and it resulted in a clear phenotype - reduced FB size, decreased *Sox9* expression - that was consistent with the role previously described for this gene during chondrogenesis and limb development^31,32^. This approach was thus used to investigate the implication of *Fgf11* and *Tbx1*. Their upregulation during FB development, confirmed by RT-PCR, flagged them as potential regulators of FB development in our transcriptome analysis. Although the knockdown of each of them individually had less impact on FB development than that of *Sox6*, the simultaneous knock-down of both resulted in a more severe developmental delay. Furthermore, this double knockdown drastically decreased the expression of both *Sox6* and *Sox9*, and reciprocally the knockdown of *Sox6* was found to decrease the expression of *Fgf11* and *Tbx1*. These results suggest *Fgf11* and *Tbx1* act in a synergistic manner, belong to the same regulatory network than *Sox6* and *Sox9*, and that their contribution to FB development involves mutual cross-regulation with these two prominent master regulators of the process. *Fgf11* encodes an intracellular FGF, a non-signaling protein known to serve as co-factor for voltage-gated sodium channels. *Fgf11* mutant mice are viable and have not been associated with any particular phenotype^33^. In contrast, mice mutant for *Tbx1*, which encodes a transcription factor, exhibit a large range of developmental anomalies, including thymus hypoplasia, abnormal cardiac outflow tract, vertebrae and cleft palate and facial structures abnormalities, but no obvious limb developmental defects^34^. However, a study in the chick embryo relying on the misexpression of a dominant-negative form of *Tbx1* found it played a role in the regulation of myogenic differentiation in the limb^35^, a defect emerging admittedly later than what we considered in our study. How can we account for the discrepancy between the mutant mouse phenotypes for *Fgf11* and *Tbx1* and those we obtained? It is possible that the use of siRNAs circumvents the activation of compensatory networks that normally buffer against deleterious mutations, as morpholinos can do in zebrafish embryos^36^. Off-targets effects remain a possibility, but there is also prior evidence of a functional link between *Tbx1* and *Sox9*, as the first has been shown to mediate the function of the second during optic capsule formation^37^ and also to regulate its expression during cranial chondrogenesis^38^. This partly explains why the Pathway Studio software identified interactions between *Tbx1* and *Sox9*, alongside others with *Fgf8* and *Fgf10*, likewise involved in regulating the formation of prechondrogenic aggregates (Supplementary Fig. S3 and Supplementary Table 5)^39–41^. Biological networks created using the same software also place *Fgf11* downstream of *Hif1α*, a factor critical to chondrocyte growth arrest and survival (Supplementary Fig. S3)^42^. Together with the experimental evidence gathered in the course of this study these data support a role for *Fgf11* and *Tbx1* in early aspects of FB development, possibly via their implication in the regulation of *Sox6* and *Sox9*.

## Supplementary information

Supplementary Figures.

Supplementary Table 1.

Supplementary Table 2.

Supplementary Table 3.

Supplementary Table 4.

Supplementary Table 5.

## Data Availability

The datasets used and analyzed during the current study are available from the corresponding author on reasonable request.
