# Supplementary material for: Whole embryo culture, transcriptomics and RNA interference identify TBX1 and FGF11 as novel regulators of limb development in the mouse
Source: Sci Rep. 2020 Feb 27;10:3597. doi: 10.1038/s41598-020-60217-w (PMC7046665; doi:10.1038/s41598-020-60217-w)
Supplement: Supplementary file 1 — Supplementary Figures. [file 41598_2020_60217_MOESM1_ESM.pdf]

Whole embryo culture, transcriptomics and RNA interference identify TBX1 and FGF11 as novel regulators of limb development in the mouse

Gautier Tejedor, Béryl Laplace-Builhé, Patricia Luz-Crawford, Said Assou, Audrey Barthelaix, Marc Mathieu, Karima Kissa, Christian Jorgensen, Jérôme Collignon, Paul Chuchana, Farida Djouad

Supplementary figure S1

A

Molecules in Network	Score	Focus Molec	Top Functions
↑**ADCYAP1R1**, APOE, APP, ↑**ARHGAP20***, ↑**CORIN***, ↑**DBC1***, ↑**DPP6**, Histone h3, HMGB1, HMOX1, ↑**HOXA2**, ↓**HOXA11***, LRP1, MBD1, ↑**MEIS1**, ↑**MYL9***, ↑**NEFL**, NPPA, PHF1, ↑**PLAT***, POLR2A, RNA polymerase II, RPS6, SFN, SLC36A4, SMAD7, ↓**SPTA1**, SRF, SUV39H1, ↑**TAGLN***, ↑**THBS2**, TNF, YAP1, ZNF217, ZNF326	23	14	Cardiovascular Disease, Cellular Assembly and Organization, Cell Death and Survival
↑**A2M**, ↑**ADRA2A**, ↑**AKAP12***, Akt, C1QBP, CD9, CELF1, EIF2A, ERK1/2, Growth hormone, GSK3A, ↑**HOXA1***, ↓**HOXD10**, ↑**IGF1***, IGFBP5, IL11, IL1RAP, INHBA, Integrin alpha 3 beta 1, IRS2, LRP1, ↑**MAGI2**, NGF, ↑**PTGER4**, RHOB, S100B, S1PR1, SLC20A1, TIMP2, ↑**TMEM64**, Vegf, VTN, ↑**WNT2B***, ↑**WT1***, ↑**ZEB2**	19	12	Cellular Movement, Embryonic Development, Cell Death and Survival
↑**AKAP12***, ASNS, ↑**ATF3***, BHLHE40, CDKN1B, CEBPD, ↑**CNN1***, CTNNB1, ↑**EGLN3***, ↓**GYPA**, HIF1A, KLF6, ↑**LOXL2***, MAP2K1/2, MGEA5, miR-34a-5p (and other miRNAs w/seed GGCAGUG), MSH2, NDRG1, ↑**PRKAR1B**, S100A6, ↑**SCN3B**, SERPINB5, SFN, SPHK1, ↑**STAB1***, ↑**SVEP1***, TGFBR2, TLR5, TLR6, TLR9, TNF, TP53, TSC1, ↑**VLDLR***, ↑**WNT2***	19	12	Cell Death and Survival, Cell Cycle, Cancer
↑**ACER2***, caspase	2	1	Lipid Metabolism, Small Molecule Biochemistry, Cell Death and Survival

B

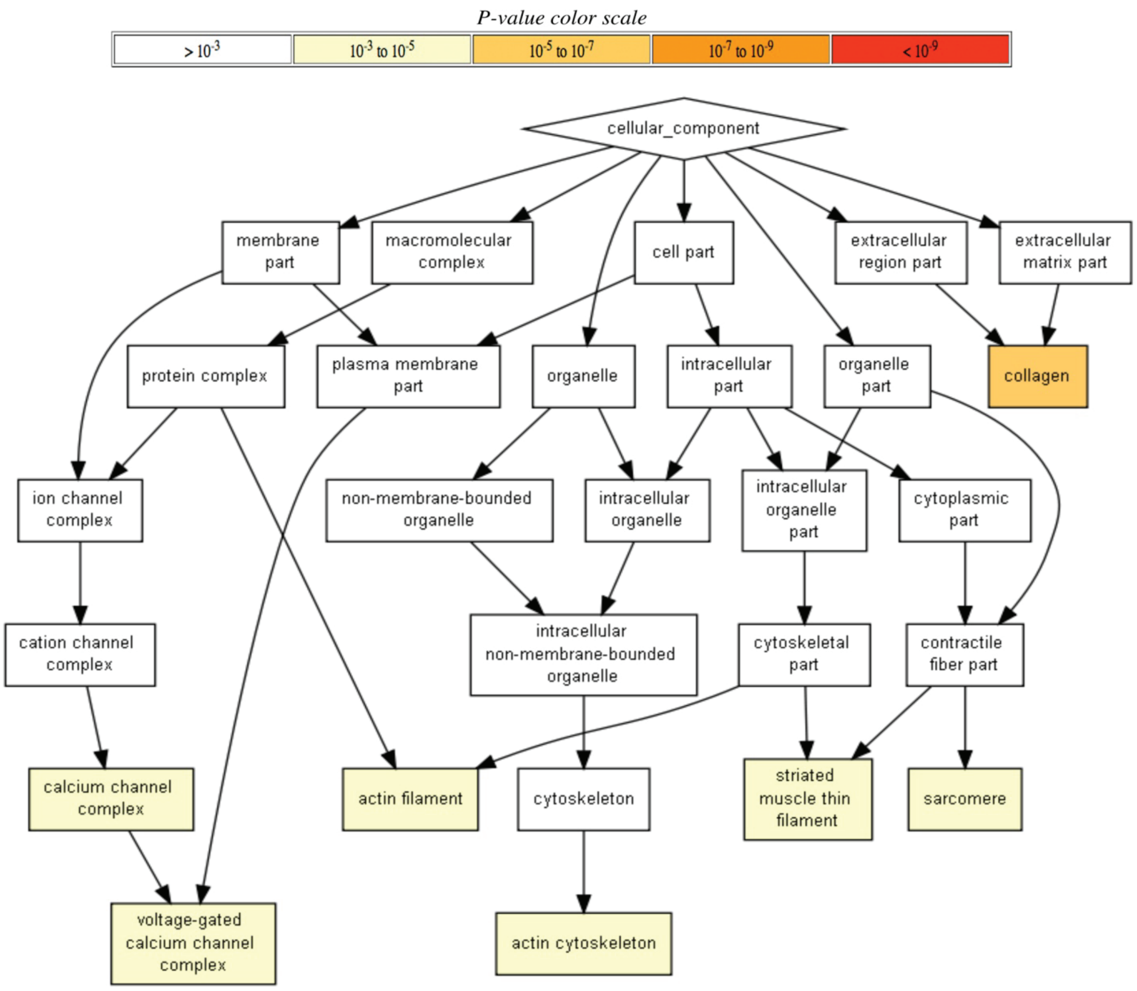

Supplementary Figure 1. (A) Overview of approach and computational analysis. The functional analyses were generated through the use of IPA (QIAGEN Inc., https://www.qiagenbioinformatics.com/products/ingenuity-pathway-analysis). During the first hours of mouse embryo culture in a controlled system, 39 out of 83 genes display a similar expression dynamic and are associated with the cell death and survival processes. (B) Gene Ontology (GO) space mapping the different lists of genes. GO terms are enriched in the annotation of cellular component highly expressed in the developing limb. Among them, the collagen, the main structural component of the connective tissues was highly differentially expressed in the *ex utero* developing limb bud as revealed by the color orange.

Supplementary figure S2

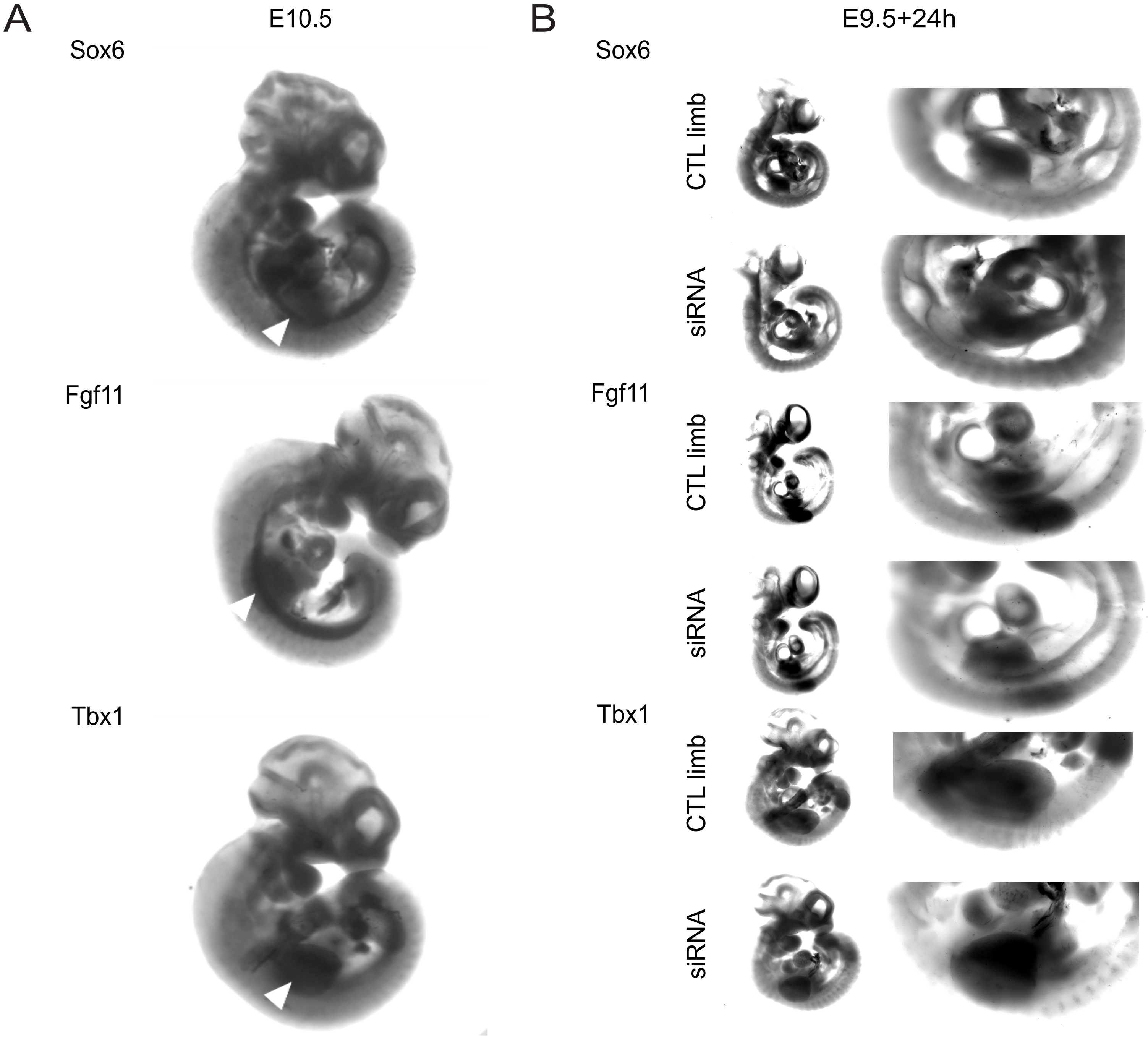

Supplementary Figure 2. (A) Representative images of *Sox6*, *Fgf11* and *Tbx1* mRNA *in situ* hybridizations performed on E10.5 embryos. Arrows indicate forelimb buds. (B) Representative images of *Sox6, Fgf11* and *Tbx1* mRNA *in situ* hybridizations performed on E9.5 embryos injected respectively with Sox6-, Fgf11- and Tbx1-siRNAs, and cultured for 24h.

Supplementary figure S3

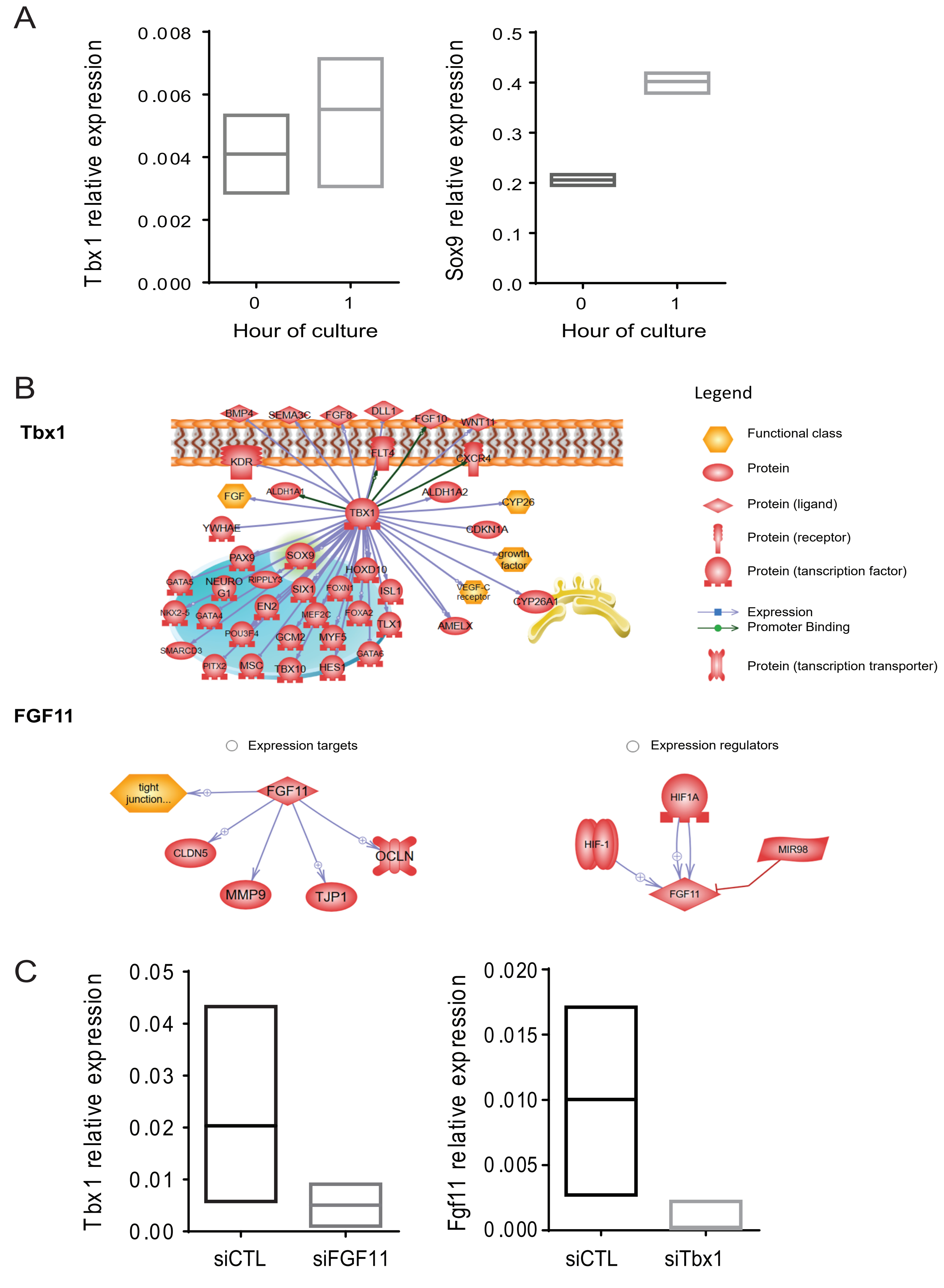

Supplementary Figure 3. (A) Relative expression profile of *Tbx1* and *Sox9* in the forelimb of E10.5 mouse embryos after 1 hour of culture. RT-qPCR on separated cells using *Rps9* as a reference gene. (B) *Tbx1* and *Fgf11* were imported into Pathway Studio and an interaction maps were created. Each node represents a gene entity of the interaction. Lines represent type of relations that were automatically extracted from the literature. *Tbx1* was related to approximately forty four different genes including *Sox9*. *Fgf11* interaction searching includes expression target and expression regulators like *hif1a*. The legend of the interaction network is summarized on the left of the figure. (C) Relative expression profile of *Tbx1* and *Fgf11* in the forelimb of E10.5 mouse embryos that have been transfected with either the siRNA control (siRNA CTL) or the siRNA against *Ffg11* or *Tbx1* (si*Fgf11* or si*Tbx1*, respectively) at the embryonic stage E9.5 and cultured 24h *ex utero*.
